# Supplementary material for: EEF1A1 deacetylation enables transcriptional activation of remyelination
Source: Nat Commun. 2020 Jul 9;11:3420. doi: 10.1038/s41467-020-17243-z (PMC7347577; doi:10.1038/s41467-020-17243-z)
Supplement: Supplementary file 1 — Supplementary Information [file 41467_2020_17243_MOESM1_ESM.pdf]

Supplementary Information

EEF1A1 Deacetylation Enables Transcriptional Activation of Remyelination

Duman et al.

Supplementary Figures 1-11

Supplementary Data 1

Supplementary Data 2

Supplementary Movie 1

Reporting Summary

Source Data

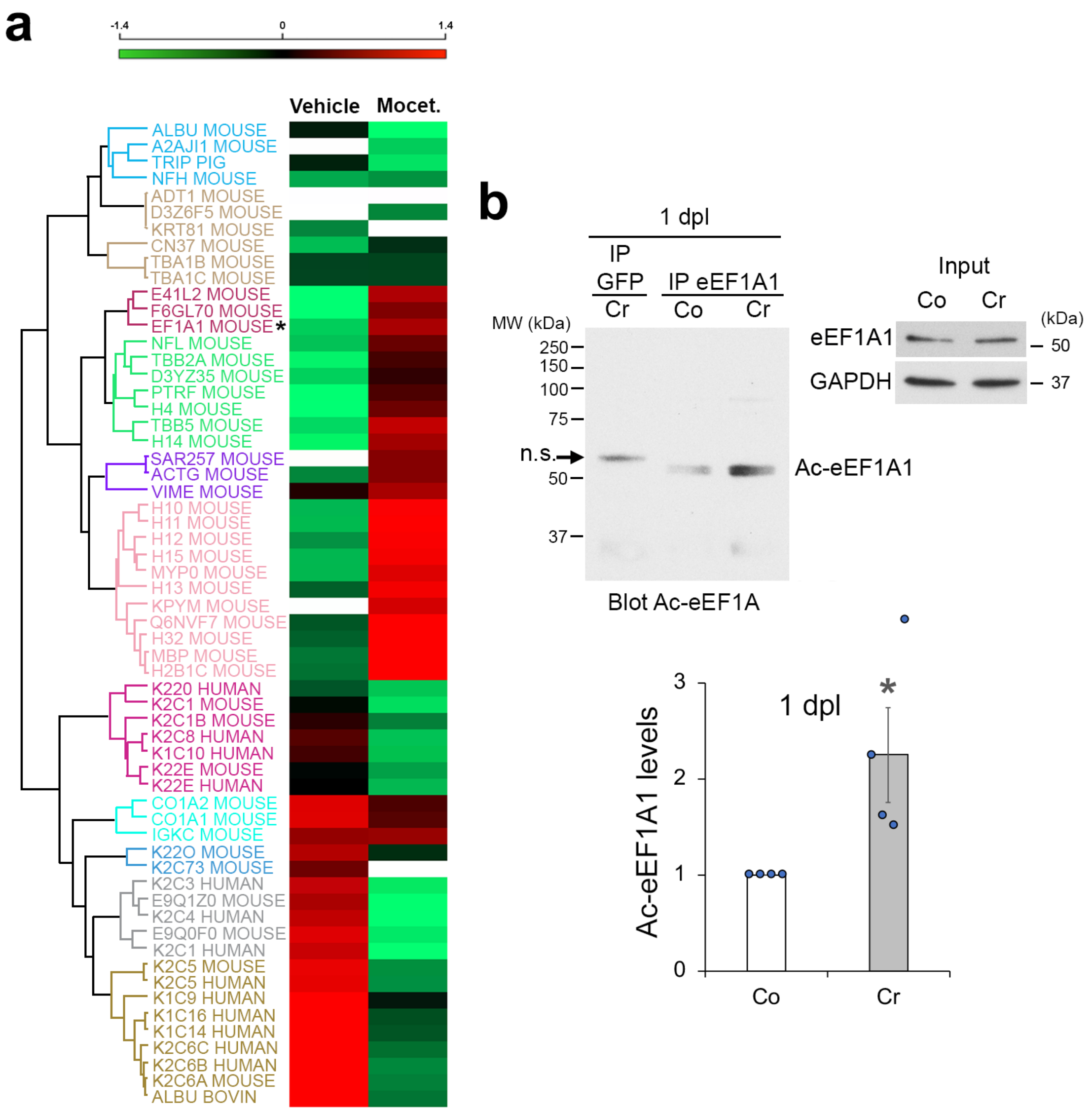

Supplementary Figure 1. eEF1A1 is acetylated after a PNS lesion and is a putative deacetylation target of HDAC1/2. (**a**) Analysis of previously reported[41] mass spectrometry by Protein Match Score Summation identifying eEF1A1 as a putative HDAC1/2 deacetylation target. Mass spectrometry was carried out on immunoprecipitates with an anti-acetylated lysine antibody. Sciatic nerves of mice treated immediately after sciatic nerve crush lesion with mocetinostat or its vehicle and collected at 1dpl were lysed and used for immunoprecipitation (IP). (**b**) IP of eEF1A1 or GFP (control) in crushed (Cr) or contralateral (Co) sciatic nerves at 1dpl and Western blot of Ac-eEF1A, and quantification of Ac-eEF1A1 levels normalized to total eEF1A1 input in crushed compared to contralateral nerves. Data are presented as mean values +/- SEM. Paired one-tailed Student's t-tests, p value: 0.042174, n=4 animals. Source data are provided as a Source Data file.

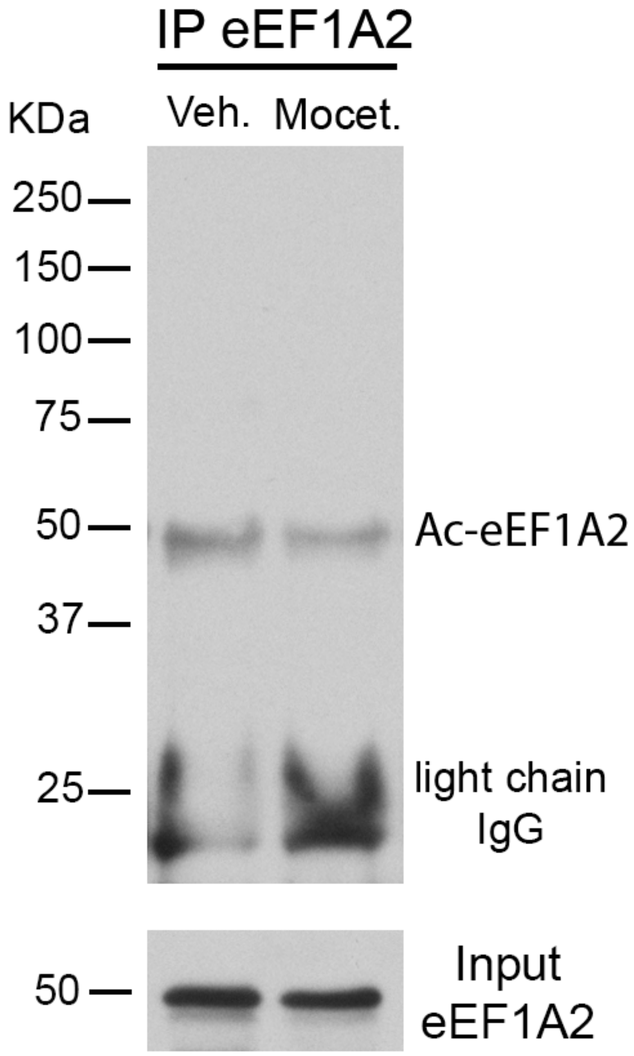

Supplementary Figure 2. eEF1A2 is not deacetylated by HDAC1/2. Denaturing immunoprecipitation (IP) of eEF1A2 and Western blot of Ac-eEF1A in SCs cultured under de-differentiating conditions and treated with the HDAC1/2 inhibitor mocetinostat (Mocet.) or its vehicle (Veh.) for 24 h, and input eEF1A2 of lysates used for IP. Representative images of 3 independent experiments are shown.

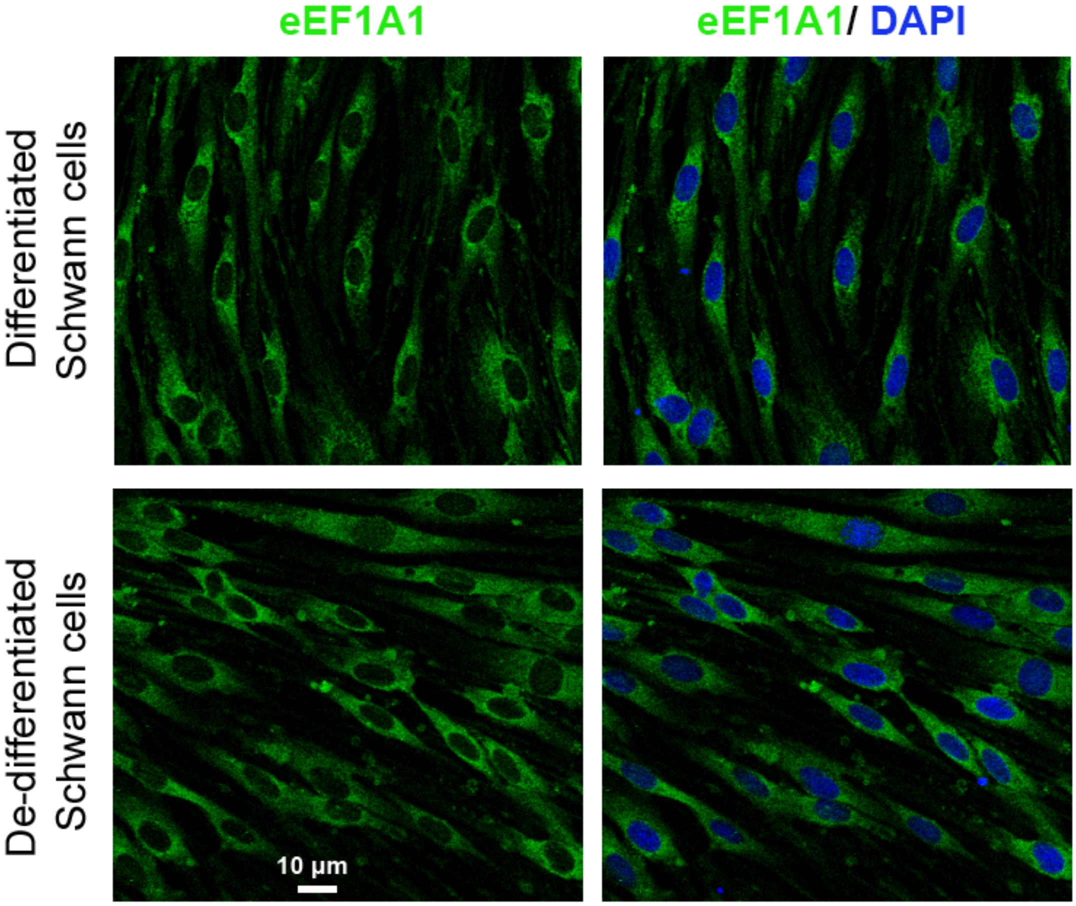

Supplementary Figure 3. eEF1A1 is an abundant protein mainly localized in the SC cytoplasm. Immunofluorescence of eEF1A1 and DAPI (nuclei) labeling in purified primary SCs cultured under differentiating and de-differentiating conditions. Representative images (single optical sections) are shown. Representative images of 3 independent experiments are shown.

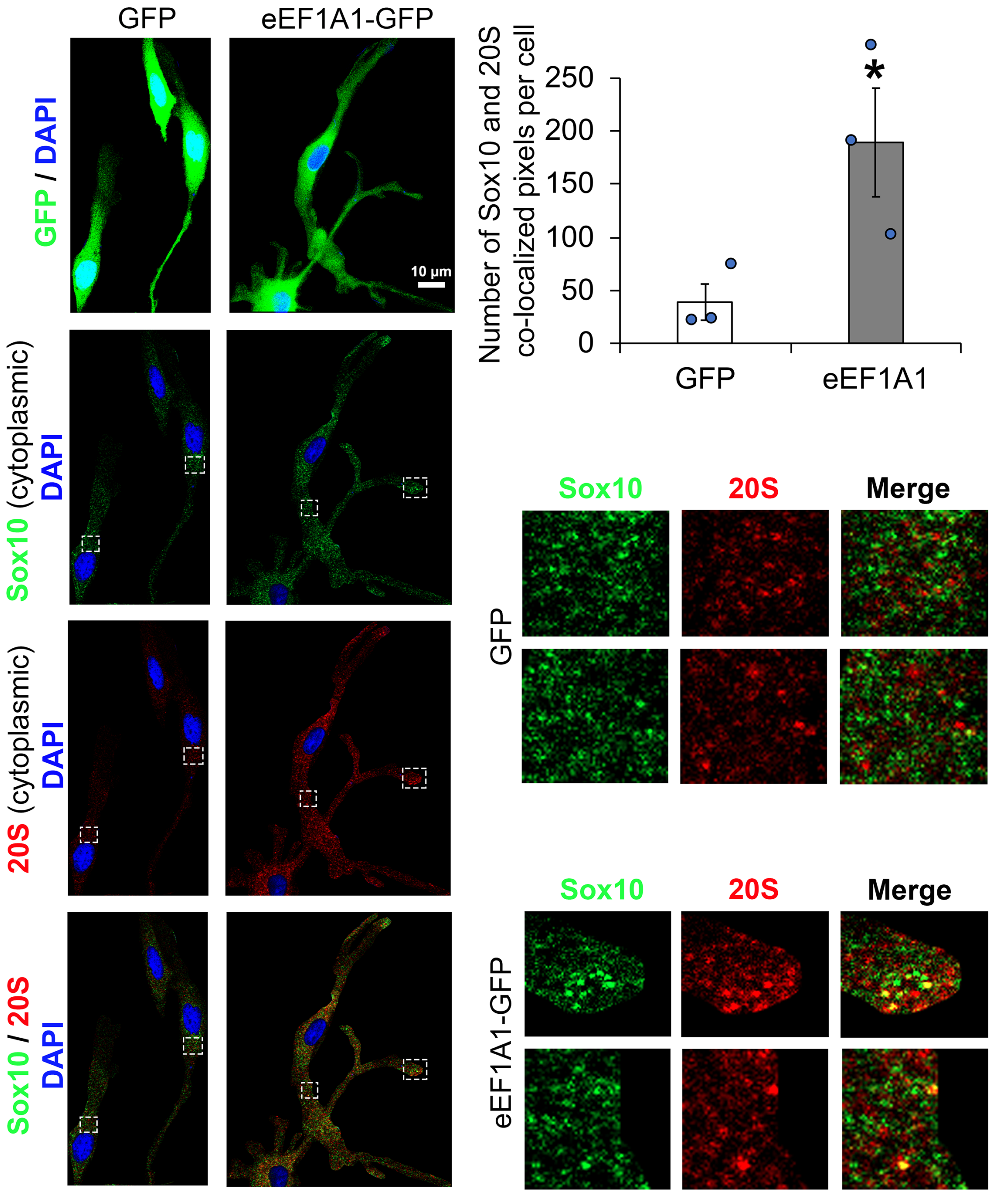

Supplementary Figure 4. eEF1A1 promotes Sox10 targeting to the proteasome. Co-immunofluorescence (confocal images, the optical section with the highest signal intensity is shown) of Sox10 (false-colored green) and the proteasomal subunit 20S (red) with GFP (green) and DAPI (blue=nuclei) labeling in SCs cultured under proliferating conditions and transfected with eEF1A1-GFP or GFP for 24 h, and quantification of co-localized (yellow) pixels of Sox10 and 20S signal per cell (the optical section with the highest signal intensity has been used for quantification). For this quantification, only transfected cells have been extracted from their original image and placed on a black background. The nuclear signal for Sox10 and 20S have been removed from the images because the high abundance of Sox10 signal in the nuclear compartment is not compatible with co-localization studies. Images on the right are magnifications of the regions highlighted by white boxes on the left images. Data are presented as mean values +/- SEM. Unpaired two-tailed Student's *t*-tests, p value: 0.048849, n=3 independent experiments, each n represents the average pixel number per cell from 3 different randomly chosen fields of view showing transfected cells, total number of quantified cells: 14 for GFP, 19 for eEF1A1-GFP. Source data are provided as a Source Data file.

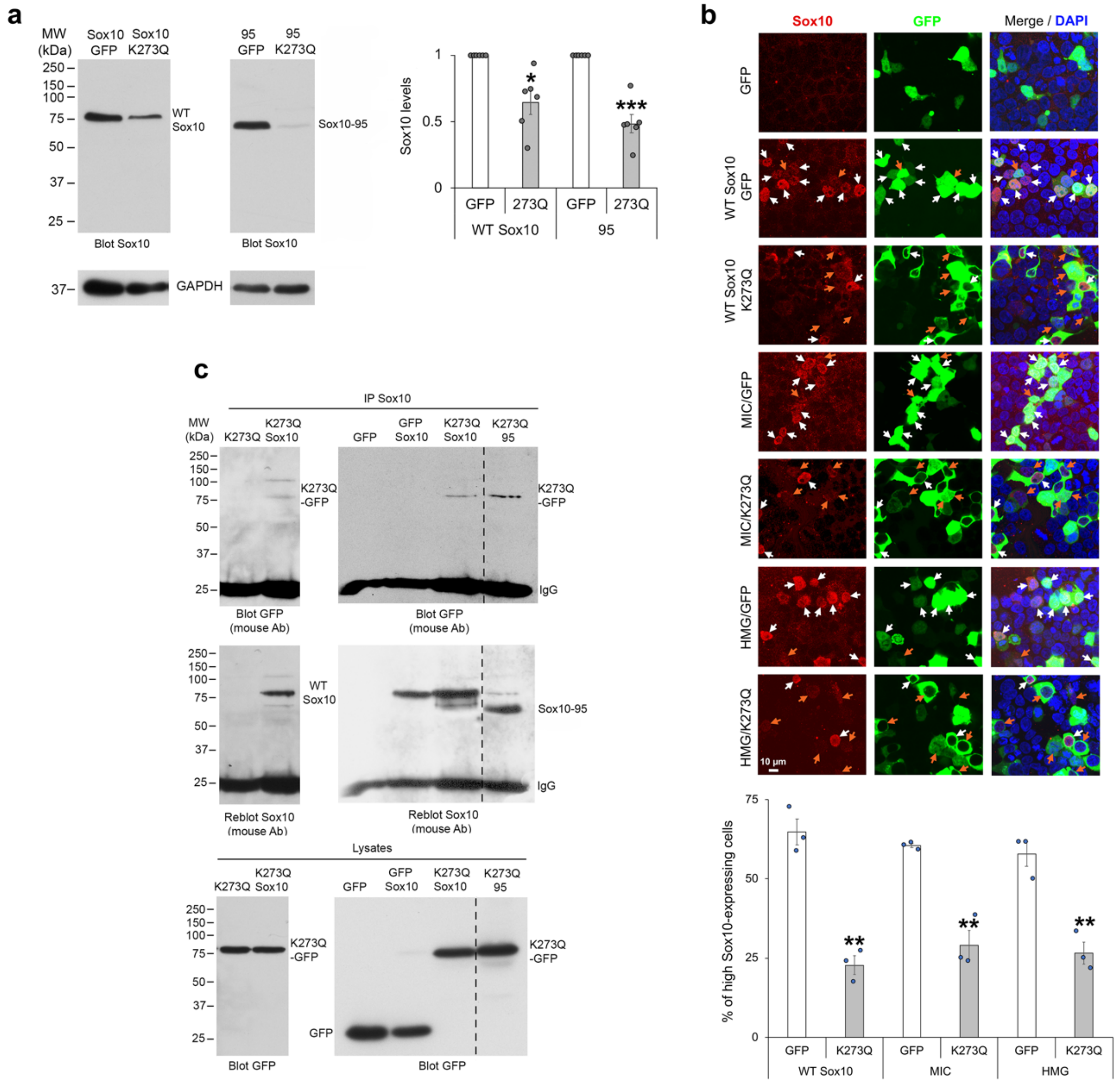

Supplementary Figure 5. The HMG domain of Sox10 is sufficient for Ac-eEF1A1 to decrease Sox10 stability. (**a**) Western blot analysis of Sox10 and GAPDH (loading control) in lysates of HEK293 cells co-transfected with wild type Sox10 or Sox10-95 together with K273Q eEF1A1 or GFP (control), and quantification of Sox10 levels normalized to GAPDH in K273Q-transfected cells as compared to the control. Data are presented as mean values +/- SEM. Paired two-tailed Student's t-tests, p values: 0.011447 (WT Sox10), 0.000651 (95), n=6 independent experiments. (**b**) Immunofluorescence of Sox10, GFP fluorescence and DAPI (nuclei) labeling in HEK293 cells transfected with GFP or co-transfected with wild type Sox10 and GFP or wild type Sox10 and K273Q or Sox10-MIC and GFP or Sox10-MIC and K273Q or Sox10-HMG and GFP or Sox10-HMG and K273Q, and percentage of high Sox10-expressing cells among transfected cells. Data are presented as mean values +/- SEM. Unpaired two-tailed Student's t-tests, p values: 0.001174 (WT Sox10), 0.002671 (MIC), 0.003994 (HMG), n=3 independent experiments, 64 to 176 transfected cells counted per n per condition. (**c**) Immunoprecipitation (IP) of Sox10 in HEK293 cells transfected with K273Q alone or GFP alone or wild type Sox10 and GFP or wild type Sox10 and K273Q or Sox10-95 and K273Q and blot GFP and Reblot Sox10. Underneath the IPs, the lysates show the inputs of GFP and K273Q-GFP. Representative images of 3 to 6 independent experiments are shown. Source data are provided as a Source Data file.

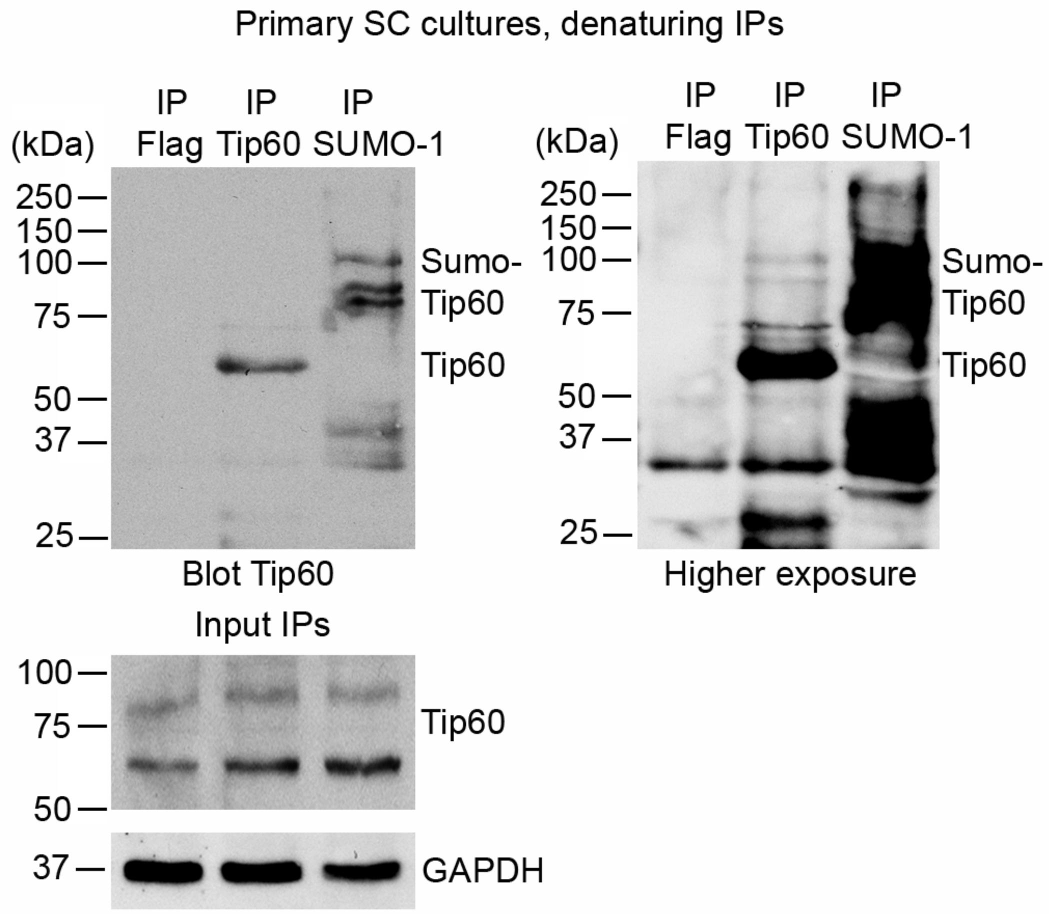

Supplementary Figure 6. Tip60 is sumoylated in primary SCs. Denaturing immunoprecipitation (IP) of SUMO-1, Tip60 or Flag (control) in primary SCs cultured under de-differentiation conditions for 1 day and blot Tip60. The images underneath the IPs show the inputs for Tip60 and GAPDH in the lysates used for the IPs. Representative images of 3 independent experiments are shown.

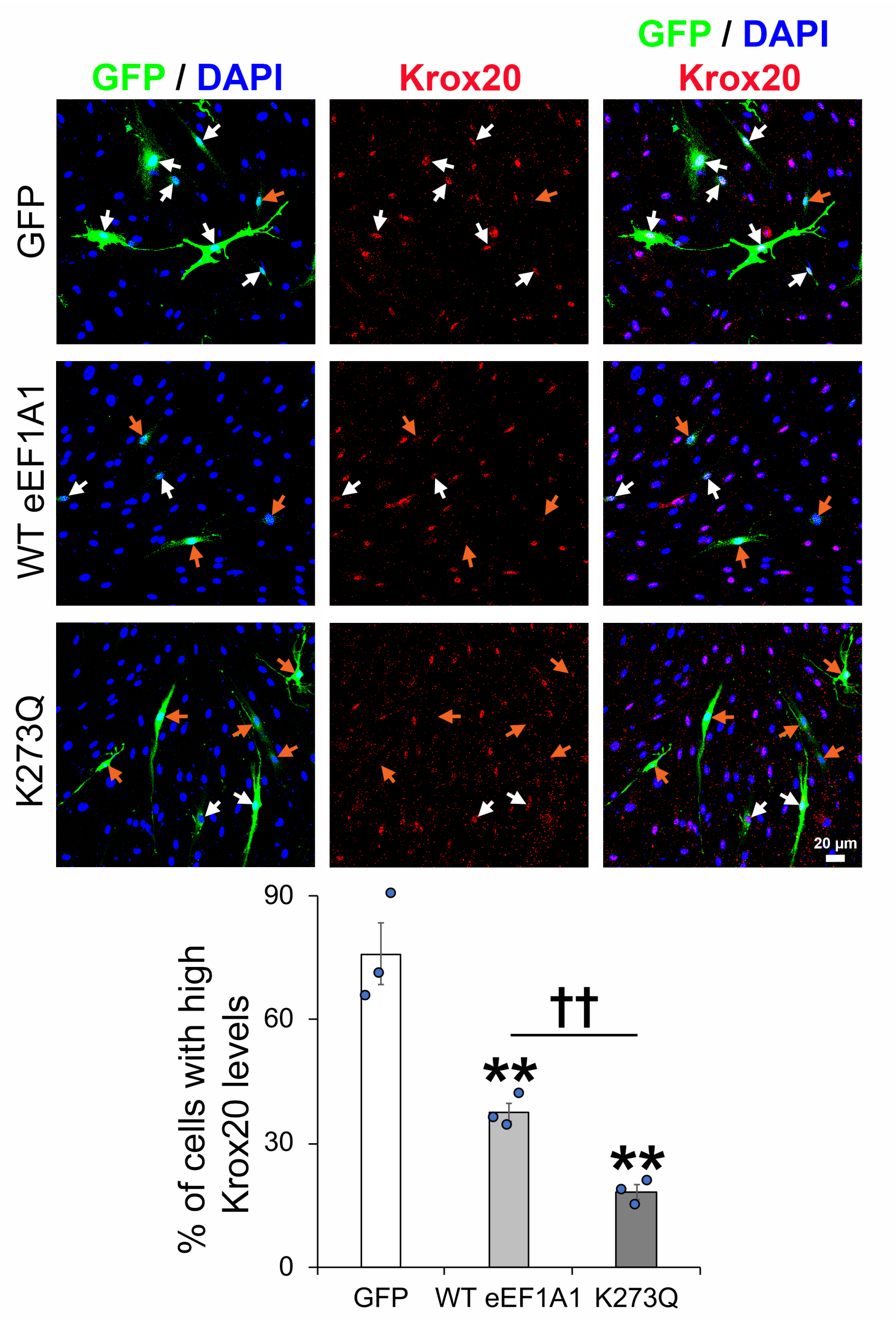

Supplementary Figure 7. Krox20 levels are further decreased in SCs expressing K273Q as compared to SCs overexpressing wild type eEF1A1. Immunofluorescence of Krox20, GFP fluorescence and DAPI (nuclei) labeling in primary SCs cultured under differentiation conditions and transfected with GFP or wild type eEF1A1 or K273Q, and percentage of high Sox10-expressing cells among transfected cells. Data are presented as mean values +/- SEM. Unpaired two-tailed Student's t-tests, p values/GFP: 0.008273 (WT eEF1A1), 0.001759 (K273Q), p value/WT-eEF1A1: 0.002657, n=3 independent experiments, 31 to 80 transfected cells counted per n per condition. Source data are provided as a Source Data file.

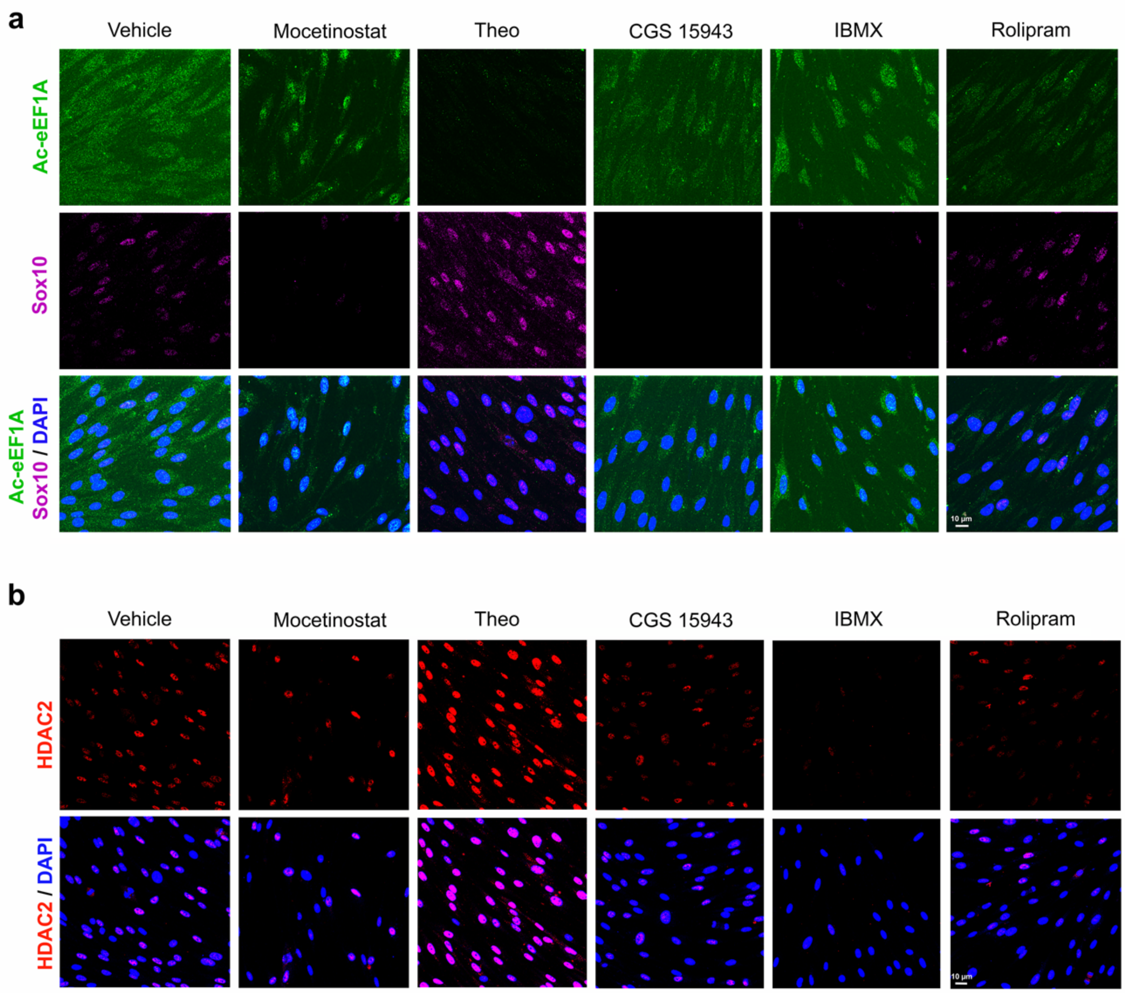

Supplementary Figure 8. Theophylline decreases Ac-eEF1A and increases Sox10 and HDAC2 levels, whereas CGS 15943, IBMX and rolipram have no effect or the opposite effect. (a, b) Co-immunofluorescence of Sox10 and Ac-eEF1A (a) or immunofluorescence of HDAC2 (b), and DAPI (nuclei) labeling in primary SCs cultured under de-differentiation conditions and treated with mocetinostat (0.6 µM) or theophylline (1 µM) or CGS 15943 (1 µM) or IBMX (500 µM) or rolipram (10 µM) or vehicle for 2 days. Representative images of 3 independent experiments are shown.

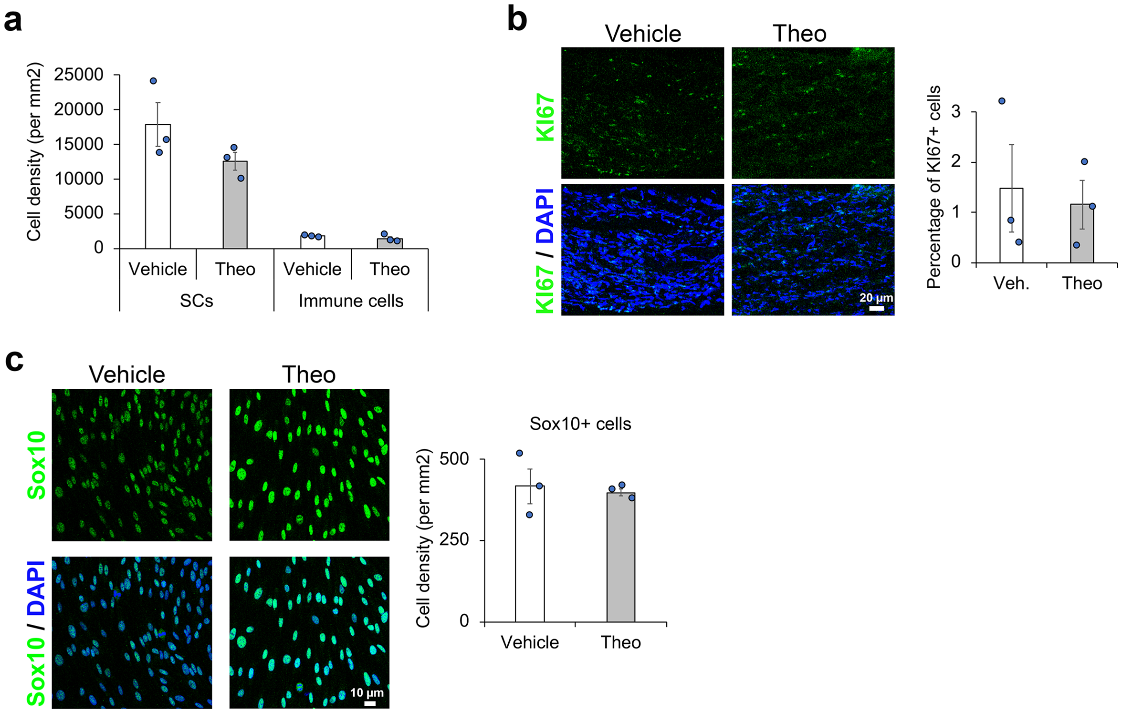

Supplementary Figure 9. Theophylline treatment does not increase cell density or proliferation. (**a**) Quantification of SC or immune cell density at 14dpl in sciatic nerves of mice treated with theophylline or vehicle for 4 days starting from 10dpl. Quantifications were done using electron microscopy images used for g ratio quantification in Fig. 5e. All SCs and immune cells imaged over 6000 to 11000 µm2 were counted per animal, n=3 animals per condition. (**b**) Immunofluorescence of KI67 (proliferating cells) and DAPI (nuclei) labeling and percentage of KI67+ cells at 12dpl in sciatic nerves of mice treated with theophylline or vehicle for 2 days starting from 10dpl. 507 to 1392 cells counted per animal, n=3 animals per condition. (**c**) Immunofluorescence of Sox10 (used as SC marker) and DAPI labeling in primary SCs cultured under dedifferentiation conditions and treated with theophylline or vehicle for 2 days, and quantification of SC density. All Sox10+ cells imaged over 40850 µm2 were counted per n per condition, n=3 independent experiments. Data are presented as mean values +/- SEM. (a-c) Unpaired one-tailed Student's t-tests, p values: 0.099934 (a, SCs), 0.166777 (a, immune cells), 0.380699 (b), 0.750254 (c). Source data are provided as a Source Data file.

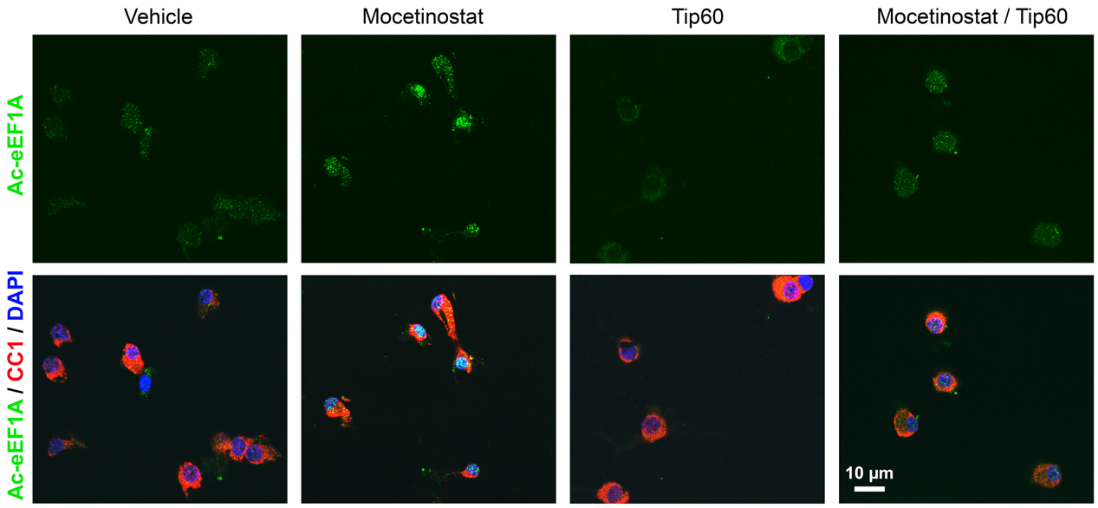

Supplementary Figure 10. Inhibition of Tip60 prevents the increase of Ac-eEF1A levels induced by the HDAC1/2 inhibitor in primary OLs. Co-immunofluorescence of Ac-eEF1A and CC1 (marker of mature OL), and DAPI (nuclei) labeling in primary OPCs differentiated in culture in OLs and treated with mocetinostat (0.6 µM) or with the specific Tip60 inhibitor TH1834 (5 µM) or with both mocetinostat and TH1834 or with vehicle for 1 day. Representative images of 3 independent experiments are shown.

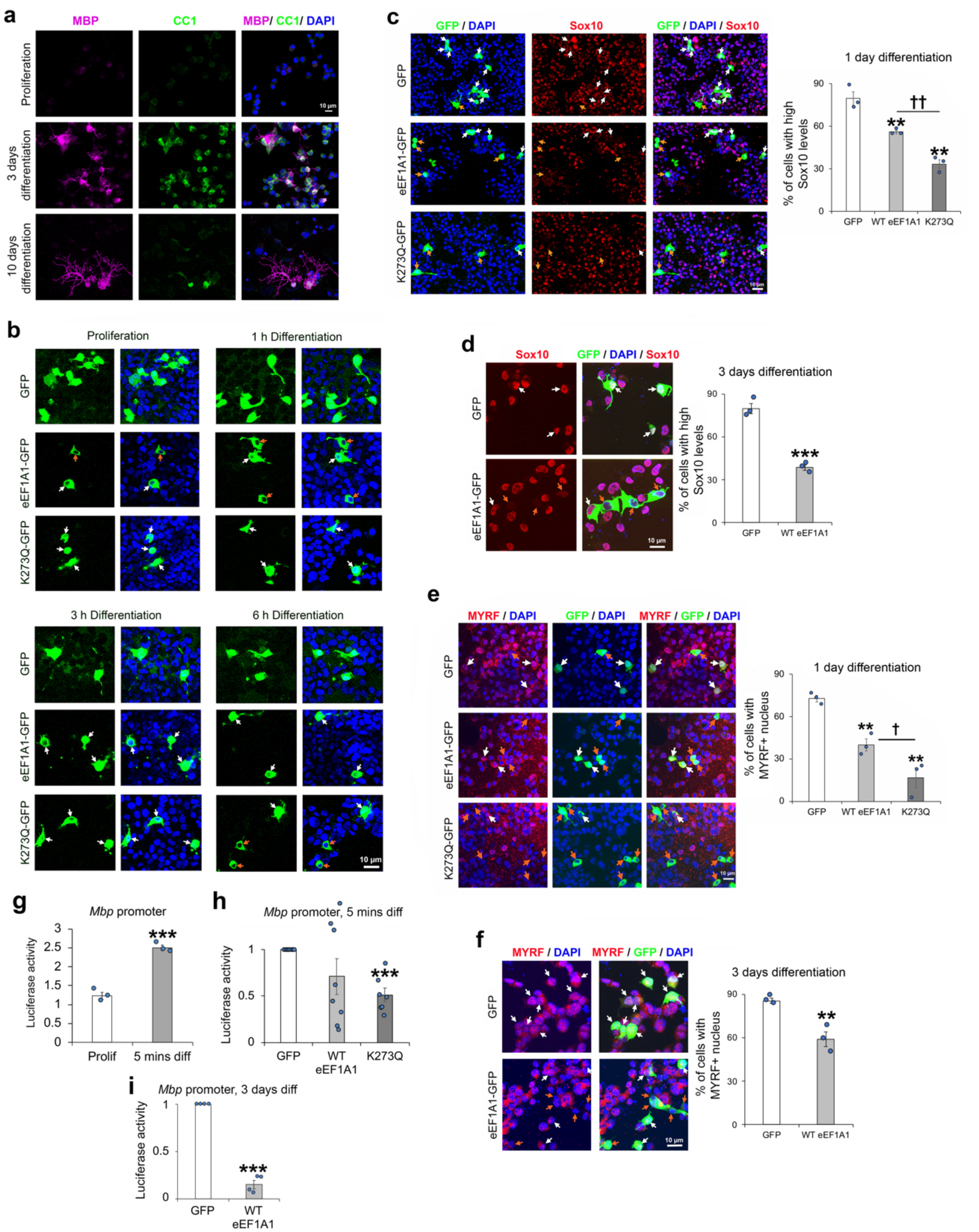

Supplementary Figure 11. K273Q eEF1A1 further decreases Sox10 and MYRF levels and the activity of the *Mbp* promoter in Oli-neu cells as compared to wild type eEF1A1. (**a**) Confocal images of MBP and CC1 co-immunofluorescence and DAPI labeling in Oli-neu cells cultured in proliferation medium or for 3 days or 10 days in differentiation medium. Single optical sections are shown. Representative images of 3 independent experiments are shown. (**b**) GFP fluorescence and DAPI labeling in Oli-neu cells cultured under proliferation conditions or under differentiation conditions for 1, 3 or 6 h. Representative images of 3 independent experiments are shown. (**c-f**) Immunofluorescence of Sox10 (c,d) or of MYRF (e,f), GFP fluorescence and DAPI (nuclei)

labeling in Oli-neu cells cultured under differentiation conditions and transfected with GFP or wild type eEF1A1 or K273Q for 1 day (c,e) or 3 days (d,f), and percentage of high Sox10-expressing cells (c,d) or of cells with nuclear MYRF (e,f) among transfected cells. White arrows indicate high Sox10-expressing cells or MYRF-positive nuclei and orange arrows low Sox10-expressing cells or MYRF-negative nuclei. (**g-i**) Quantification of *Mbp* promoter activity (by luciferase gene reporter assay) in Oli-neu cells (g) cultured for 24 h in proliferation medium (Prolif) or for 23 h 55 min + 5 min in differentiation medium (5 mins diff), or (h) transfected for 24 h with wild type eEF1A1 or K273Q as compared to GFP-transfected cells and induced to differentiate for 5 min, or (i) transfected for 24 h with wild type eEF1A1 as compared to GFP-transfected cells at 2 days of differentiation and collected at 3 days of differentiation (3 days diff). Data are presented as mean values +/- SEM. Unpaired (c-e) or paired (f) two-tailed Student's t-tests, p values: 0.008984 (c, WT eEF1A1/GFP), 0.001308 (c, K273Q/GFP), 0.002528 (c, K273Q/WT eEF1A1), 0.000582 (d), 0.002513 (e, WT eEF1A1/GFP), 0.001663 (e, K273Q/GFP), 0.049454 (K273Q/WT eEF1A1), 0.00797 (f), 0.000423 (g), 0.086291 (h, WT eEF1A1/GFP), 0.000551 (h, K273Q/GFP), 0.00031 (i). (c, d, e) n=3, (d) n=7-8. (c-f) 18 to 64 transfected cells quantified per n. Source data are provided as a Source Data file.
