# Supplementary material for: EEF1A1 deacetylation enables transcriptional activation of remyelination
Source: Nat Commun. 2020 Jul 9;11:3420. doi: 10.1038/s41467-020-17243-z (PMC7347577; doi:10.1038/s41467-020-17243-z)
Supplement: Supplementary file 3 — Description of Additional Supplementary Files [file 41467_2020_17243_MOESM3_ESM.docx]

Description of Additional Supplementary Files

**Title:** Supplementary Data 1.

**Description:** Mass spectrometry analysis of eEF1A1 acetylated peptides in mocetinostat-treated SCs as compared to vehicle-treated SCs. Detection of eEF1A1 acetylated peptides after eEF1A1 IP in lysates of SCs treated with mocetinostat (0.6 µM) or vehicle for 24 h.

**Title:** Supplementary Data 2.

**Description:** Mass spectrometry analysis of eEF1A1 putative binding partners at 2dpl compared to unlesioned conditions in mouse sciatic nerves. Experiment A = IP GFP (control) in unlesioned adult mouse sciatic nerves, Experiment B = IP eEF1A1 in unlesioned adult mouse sciatic nerves, Experiment C = IP GFP at 2dpl in adult mouse sciatic nerves, Experiment D = IP eEF1A1 at 2dpl in adult mouse sciatic nerves. Six unlesioned and 6 lesioned (collected at 2dpl) mouse sciatic nerves have been pooled separately (1 pool of unlesioned sciatic nerves and 1 pool of lesioned sciatic nerves); each pool has been divided into 2 equal volumes, one for IP with the GFP antibody and one for IP with the eEF1A1 antibody. Fifteen microgrammes of antibody were used per IP.

**Title:** Supplementary Movie 1.

**Description:** Sox10 is targeted to the proteasome in de-differentiated SCs. 3D reconstruction after confocal imaging of Sox10 (green) and the 20S proteasome subunit (red) co-immunofluorescence in de-differentiated SC.
