# Supplementary material for: EEF1A1 deacetylation enables transcriptional activation of remyelination
Source: Nat Commun. 2020 Jul 9;11:3420. doi: 10.1038/s41467-020-17243-z (PMC7347577; doi:10.1038/s41467-020-17243-z)
Supplement: Supplementary file 7 — Reporting Summary [file 41467_2020_17243_MOESM7_ESM.pdf]

nature research

Corresponding author(s): Claire Jacob

Last updated by author(s): Jun 11, 2020

Reporting Summary

Nature Research wishes to improve the reproducibility of the work that we publish. This form provides structure for consistency and transparency in reporting. For further information on Nature Research policies, see Authors & Referees and the Editorial Policy Checklist .

Statistics

For all statistical analyses, confirm that the following items are present in the figure legend, table legend, main text, or Methods section.

n/a	Confirmed	
☐	☒	The exact sample size (*n*) for each experimental group/condition, given as a discrete number and unit of measurement
☐	☒	A statement on whether measurements were taken from distinct samples or whether the same sample was measured repeatedly
☐	☒	The statistical test(s) used AND whether they are one- or two-sided<br>*Only common tests should be described solely by name; describe more complex techniques in the Methods section.*
☒	☐	A description of all covariates tested
☒	☐	A description of any assumptions or corrections, such as tests of normality and adjustment for multiple comparisons
☐	☒	A full description of the statistical parameters including central tendency (e.g. means) or other basic estimates (e.g. regression coefficient) AND variation (e.g. standard deviation) or associated estimates of uncertainty (e.g. confidence intervals)
☐	☒	For null hypothesis testing, the test statistic (e.g. *F*, *t*, *r*) with confidence intervals, effect sizes, degrees of freedom and *P* value noted<br>*Give P values as exact values whenever suitable.*
☒	☐	For Bayesian analysis, information on the choice of priors and Markov chain Monte Carlo settings
☒	☐	For hierarchical and complex designs, identification of the appropriate level for tests and full reporting of outcomes
☒	☐	Estimates of effect sizes (e.g. Cohen's *d*, Pearson's *r*), indicating how they were calculated

Our web collection on statistics for biologists contains articles on many of the points above.

Software and code

Policy information about availability of computer code

Data collection	No sofware was used for data collection
Data analysis	MaxQuant Software version 1.4.1.2, Fiji version 1.0, Microsoft Excel for Mac version 16.34, Adobe Photoshop CC 20.0.8 Release

For manuscripts utilizing custom algorithms or software that are central to the research but not yet described in published literature, software must be made available to editors/reviewers. We strongly encourage code deposition in a community repository (e.g. GitHub). See the Nature Research guidelines for submitting code & software for further information.

Data

Policy information about availability of data

All manuscripts must include a data availability statement. This statement should provide the following information, where applicable:

- Accession codes, unique identifiers, or web links for publicly available datasets
- A list of figures that have associated raw data
- A description of any restrictions on data availability

Data availability

All numerical data are available as a Source file (associated Figures: Figs. 1-5 and 7-9, Supplementary Figs. 1, 4, 5, 7, 9, 11). The mass spectrometry proteomics data have been deposited to the ProteomeXchange Consortium via the PRIDE [1] partner repository with the dataset identifiers PXD010552 [http://proteomecentral.proteomexchange.org/cgi/GetDataset?ID=PXD010552], PXD005383 [http://proteomecentral.proteomexchange.org/cgi/GetDataset?ID=PXD005383] and PXD017579 [http://proteomecentral.proteomexchange.org/cgi/GetDataset?ID=PXD017579]. There is no restriction on data availability.

Field-specific reporting

Please select the one below that is the best fit for your research. If you are not sure, read the appropriate sections before making your selection.

- [x] Life sciences
- [ ] Behavioural & social sciences
- [ ] Ecological, evolutionary & environmental sciences

For a reference copy of the document with all sections, see nature.com/documents/nr-reporting-summary-flat.pdf

Life sciences study design

All studies must disclose on these points even when the disclosure is negative.

Sample size	Sample size was determined by the minimal number of animals or individual experiment required to obtain statistically significant results and increased in some cases to improve confidence in the results obtained.
Data exclusions	No animal or data point was excluded from the analysis.
Replication	All data presented were successfully reproduced. For each data set presented, experiments were performed at least 3 times independently or at least 3 animals were used.
Randomization	For animal treatment, we used stratified random allocation by blocks with the strata gender, age and weight. To minimize heterogeneity between groups, we used littermates of treated (Theophylline or Mocetinostat) animals as vehicle-treated controls.
Blinding	The experimenter collecting data (e.g. behavioral analyses) or analyzing data (e.g. Immunofluorescence) was blinded to the treatment and received only the animal number given at birth by the animal caretaker. Treatment allocation and collection/analysis of data were done by different persons.

Reporting for specific materials, systems and methods

We require information from authors about some types of materials, experimental systems and methods used in many studies. Here, indicate whether each material, system or method listed is relevant to your study. If you are not sure if a list item applies to your research, read the appropriate section before selecting a response.

Materials & experimental systems

n/a	Involved in the study	
	x	Antibodies
	x	Eukaryotic cell lines
x		Palaeontology
	x	Animals and other organisms
x		Human research participants
x		Clinical data

Methods

n/a	Involved in the study	
x		ChIP-seq
x		Flow cytometry
x		MRI-based neuroimaging

Antibodies

Antibodies used	All antibodies are described in the Methods section, along with the name of the manufacturer, the catalog number, the concentration used and the last lot number used. Primary antibodies: eEF1A1 (abcam, cat. # ab157455, lot # GR231741-13), eEF1A-pan (Acetyl-Lys41) (Labforce/AssaybioTech, cat. # D12106, lot # 410212106), HDAC2 (Santa Cruz Biotechnology, cat. # sc-7899, lot # E0912), HDAC2 (Sigma, cat. # H2663, lot # 096M4799V), Sox10 (DCS Innovative Diagnostik-Systeme, cat. # SI058C01, lot # S294), Sox10-Nterm (kindly provided by Dr. Michael Wegner, University of Erlangen), Sox10 (abcam, cat. # ab216020, lot # GR3272630-2), CC1 (Calbiochem, cat. # OP80, lot # 2869730), Olig2 (R&D systems, cat. # AF2418, lot # UPA0512081), 20S proteasome subunit (abcam, cat. #ab22674, lot # GR283474-6), MBP (BIO-RAD, cat. #MCA409S, lot # 161031A), S100 (GeneTex, cat. # GTX11179, lot # 821503399), F4/80 (Lucernachem, cat. # GTX26640, lot # 821403776), GFP (abcam, cat # ab290, lot # GR196475-1), GFP (Origene, cat. # TA150041, lot # W002), GAPDH (Genetex, cat. # GTX28245, lot # 821705388), P0 (cAves Labs, cat. # PZO, lot # PZO0308), Krox20 (provided by Dr. Dies Meijer, University of Edinburgh), Lamin A/C (Sigma, cat. # SAB4200236, lot # 055M4822V), KAT5/Tip60 (abcam, cat. #ab151432, lot # GR113712-14), STAT3 (abcam, cat. #ab119352, lot # GR306889-4), EEF1A2 (proteintech, cat. # 16091-1-AP, lot # 1370), Flag (Sigma, cat. # F1804, lot # SLBM0089V), SUMO-1 (Santa-Cruz, cat. # sc-5308, lot # F-1913).<br>All secondary antibodies were from Jackson ImmunoResearch.
Validation	- eEF1A1 (abcam, cat. # ab157455, lot # GR231741-13), validated by the company by Western blot on rat, mouse and human eEF1A1, by immunoprecipitation on human eEF1A1, by immunocytochemistry on human cells, by immunohistochemistry on human tissues, by flow cytometry on human cells.<br>- eEF1A-pan (Acetyl-Lys41) (Labforce/AssaybioTech, cat. # D12106, lot # 410212106), validated by the company by Western blot and ELISA on human, mouse and rat protein.<br>- HDAC2 (Santa Cruz Biotechnology, cat. # sc-7899, lot # E0912), validated by 127 citations (for example, Choi, H.K. et al., 2015,

Nat Commun. 6: 7390 ; Brügger, V. et al., 2017, Nat Commun. 8:14272 ; Steger, D.J. el al., 2010, Genes Dev. 24:1035-1044 ; Hang, C.T. et al., 2010, Nature 466:62-67) by immunoprecipitation, Western blot, immunocytochemistry and immunohistochemistry on human, mouse and rat.
- HDAC2 (Sigma, cat. # H2663, lot # 096M4799V), validated by the company by Western blot, immunoprecipitation, immunohistochemistry, ELISA on rat, canine, human, chicken, mouse, bovine.
- Sox10 (DCS Innovative Diagnostik-Systeme, cat. # SI058C01, lot # S294), validated by the company by immunohistochemistry on human tissues and by Brügger, V. et al., 2017, Nat Commun. 8:14272, by Western blot and immunoprecipitation on mouse and rat Sox10.
- Sox10-Nterm (kindly provided by Dr. Michael Wegner, University of Erlangen, self-made) validated by Michael's Wegner lab by electrophoretic mobility supershift assay and Western blot on rat and human Sox10, and validated by our group by immunofluorescence on rat Sox10.
- Sox10 (abcam, cat. # ab216020, lot # GR3272630-2), validated by the company by immunohistochemistry and Western blot on human and mouse Sox10 and by De Logu, F. et al., 2017, Nat Commun. 8:1887 by immunohistochemistry on mouse tissues.
- CC1 (Calbiochem, cat. # OP80, lot # 2869730), validated by the company by immunocytochemistry and immunohistochemistry on mouse, rat and human CC1.
- Olig2 (R&D systems, cat. # AF2418, lot # UPA0512081), validated by the company by Western blot, immunocytochemistry, immunohistochemistry on rat, mouse and human Olig2.
- 20S proteasome subunit (abcam, cat. #ab22674, lot # GR283474-6), validated by the company by immunocytochemistry, Western blot, ELISA, flow cytometry on Mouse, Rat, Rabbit, Human, Saccharomyces cerevisiae, Potato.
- MBP (BIO-RAD, cat. #MCA409S, lot # 161031A), validated by the company by Western blot, immunofluorescence, ELISA, radioimmunoassay on Mouse, Rabbit, Mammals, Rat, Guinea Pig, Sheep, Human, Chicken, Pig.
- S100 (GeneTex, cat. # GTX11179, lot # 821503399), validated by the company by immunocytochemistry, immunohistochemistry and ELISA on Human, Rat, Rabbit, Goat, Sheep, Bovine, Cat, Dog, Pig.
- F4/80 (Lucernachem, cat. # GTX26640, lot # 821403776), validated by the company by Western blot, immunocytochemistry, immunohistochemoistry, FACS, immunoprecipitation radioimmunoassay on mouse and human F4/80.
- GFP (abcam, cat # ab290, lot # GR196475-1), validated by the company by Western blot, immunoprecipitation, immunocytochemistry, immunohistochemistry, ELISA, flow cytometry.
- GFP (Origene, cat. # TA150041, lot # W002), validated by the company by immunofluorescence and Western blot.
- GAPDH (Genetex, cat. # GTX28245, lot # 821705388), validate by the company by Western blot, Immunocytochemistry, immunoprecipitation on Human, Mouse, Rat, Rabbit, Cat, Dog, Pig, Fish.
- P0 (Aves Labs, cat. # PZO, lot # PZO0308), validated by the company by Western blot and immunohistochemistry on human, mouse and rat P0, and by Brügger, V. et al., 2017, Nat Commun. 8:1887 by immuno-electron microscopy on mouse tissues.
- Krox20 (provided by Dr. Dies Meijer, University of Edinburgh, self-made), validated by the Meijer group by immunohistochemistry on mouse on mouse tissues (Darbas A. et al., 2004, Dev Biol. 21:4612-4620) and by our group by Western blot on mouse tissues and rat cells.
- Lamin A/C (Sigma, cat. # SAB4200236, lot # 055M4822V), validated by the company by Western blot, immunoprecipitation and immunofluorescence on human, mouse, monkey, bovine, canine, rat, hamster Lamin A/C.
- KAT5/Tip60 (abcam, cat. #ab151432, lot # GR113712-14), validated by the company by Western blot and immunohistochemistry on human Tip60.
- STAT3 (abcam, cat. #ab119352, lot # GR306889-4), validated by the company by Western blot, immunocytochemistry, immunohistochemistry, immunoprecipitation, flow cytometry on Human, mouse, monkey and rat Stat3.
- EEF1A2 (proteintech, cat. # 16091-1-AP, lot # 1370), validated by the company by Western blot, immunocytochemistry, immunohistochemistry, immunoprecipitation, ELISA on human, mouse, rat, oysters, zebrafish.
- Flag (Sigma, cat. # F1804, lot # SLBM0089V), validated by the company and citations (for example, Jahn A. et al., 2017, EMBO Rep. 18:929-946 ; Mignogna M.L. et al., 2015, Nat Commun., 6:6504 ; Kim Y-C. et al., 2019, Nat Chem Biol. 15:907-916) by Western blot, immunoprecipitation, immunofluorescence.
- SUMO-1 (Santa-Cruz Biotechnology, cat. # sc-5308, lot # F-1913), validated by the company by Western blot, immunoprecipitation, immunofluorescence and immunohistochemistry on human, mouse and rat SUMO-1.

Eukaryotic cell lines

Policy information about cell lines

Cell line source(s)	We used HEK293T cells from ATCC and Oli-neu cells, which were generated in the lab of Prof. Dr. Jacqueline Trotter, University of Mainz, Germany (Ref. 55 in the manuscript: Jung et al., Eur. J. Neurosci., 1995). We used also primary rat Schwann cells and primary rat oligodendrocytes that we collected and purified ourselves.
Authentication	Identity and purity of our primary Schwann cells were checked for each primary preparation by immunofluorescence of Schwann cell-specific markers (p75, Sox10, Oct6, Krox20, P0, MAG). Identity and purity of our primary oligodendrocyte cultures were checked on each preparation by immunofluorescence of Sox10 and CC1. Oli-neu cells were authenticated by immunofluorescence of oligodendrocyte-specific markers (Olig2, Sox10, CC1, MBP). HEK293T cells were authenticated by their ability to package lentiviruses.
Mycoplasma contamination	Mycoplasma contamination was not tested, because of the low incidence of mycoplasma contamination in primary cells, and because mycoplasma contamination results in inefficiency of transfection, which we did not observe in our primary cells or in HEK293T cells or Oli-neu cells.
Commonly misidentified lines (See ICLAC register)	No commonly misidentified cell lines were used in this study.

Animals and other organisms

Policy information about studies involving animals; ARRIVE guidelines recommended for reporting animal research

Laboratory animals	Male and female mice (mixed strains back-crossed at least 10 times to C57BL/6J) were used. Three age groups were used: pups, P1-4; young adults, 3-4-month old; aged mice, 18-month old. Mice were housed in a standard mouse facility with controlled ventilation (inward airflow, exhaust to the outside), temperature (22±2°C) and humidity (65±5%) in individually ventilated type II long cages (L 365 mm x B 207 mm x H 140 mm) containing sawdust bedding, a cardboard cylinder and 2 paper tissues on the floor, food pellet and water ad libitum. Light cycle: 12:12h.
Wild animals	The study did not involve wild animals.
Field-collected samples	The study did not involve samples collected from the field.
Ethics oversight	Veterinary office of the Canton of Fribourg, Switzerland and Veterinary office (Landesuntersuchungsamt) of Rheinland-Pfalz, Germany

Note that full information on the approval of the study protocol must also be provided in the manuscript.
